# Isotactic and Syndiotactic Alternating Ethylene/Propylene Copolymers Obtained Through Non-Catalytic Hydrogenation of Highly Stereoregular *cis*-1,4 Poly(1,3-diene)s

**DOI:** 10.3390/molecules22050755

**Published:** 2017-05-06

**Authors:** Giovanni Ricci, Antonella Caterina Boccia, Giuseppe Leone, Ivana Pierro, Giorgia Zanchin, Miriam Scoti, Finizia Auriemma, Claudio De Rosa

**Affiliations:** 1 CNR-Istituto per lo Studio delle Macromolecole (ISMAC), Via A. Corti 12, I-20133 Milano, Italy; antonella.boccia@ismac.cnr.it (A.C.B.); giuseppe.leone@ismac.cnr.it (G.L.); ivana.pierro@ismac.cnr.it (I.P.); giorgia.zanchin@ismac.cnr.it (G.Z.); 2 Dipartimento di Scienze Chimiche, Università di Napoli Federico II, Complesso Monte S. Angelo, Via Cintia, I-80126 Napoli, Italy; miriam.scoti@unina.it (M.S.); auriemma@unina.it (F.A.); claudio.derosa@unina.it (C.D.R.); 3 Dipartimento di Chimica, Università degli Studi di Milano, via C. Golgi 19, I-20133 Milano, Italy

**Keywords:** ethylene/propylene copolymers, hydrogenation, NMR, XRD

## Abstract

The homogeneous non-catalytic hydrogenation of *cis*-1,4 poly(isoprene), isotactic *cis*-1,4 poly(1,3-pentadiene) and syndiotactic *cis*-1,4 poly(1,3-pentadiene) with diimide, formed by thermal decomposition of *para*-toluenesulfonylhydrazide, is examined. Perfectly alternating ethylene/propylene copolymers having different tacticity (i.e., isotactic and syndiotactic), which are difficult to synthesize by stereospecific copolymerization of the corresponding monomers, are obtained. Both isotactic and syndiotactic alternating ethylene/propylene copolymers are amorphous, with very low glass transition temperatures.

## 1. Introduction

Nowadays ethylene/propylene (E/P) copolymers are one of the most important families of polymeric materials, with endless applications. The performance of E/P copolymers can be easily tuned by varying the comonomer composition, comonomer distribution and chain stereoregularity. When the comonomers are randomly distributed, amorphous polymers (E/P rubbers and E/P/diene rubbers), emerging as a new class of thermoplastic elastomers, can be obtained [1]. If the comonomer is isolated, crystalline copolymers result, which are widely used as impact-strength modifiers in blends with isotactic poly(propylene) [2].

The synthesis of alternating E/P copolymers has been one of the challenging subjects of both practical and fundamental interest in recent years [3]. The development of metallocene [4], and post-metallocene complexes [5] for olefin polymerization has opened new opportunities for the synthesis of alternating copolymers with controlled microstructure and properties. Alternating E/P copolymers have been obtained by the copolymerization of ethylene with propylene with some ansa-zirconocenes-based catalysts [6,7,8], and by living polymerization of 1-pentene with an α-diimine Ni(II) catalyst through a controlled chain-walking mechanism [9].

**Scheme 1:**
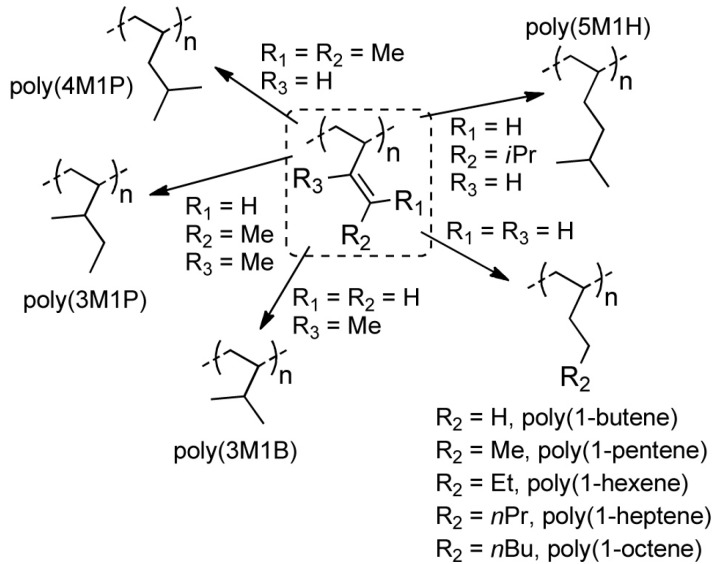
Saturated α-olefin polymers which, at least in principle, can be obtained by hydrogenation of poly(1,3-diene)s with a 1,2 structure (4M1P = 4-methyl-1-pentene, 3M1P = 3-methyl-1-pentene, 5M1H = 5-methyl-1-hexene, 3M1B = 3-methyl-1-butene).

**Scheme 2:**
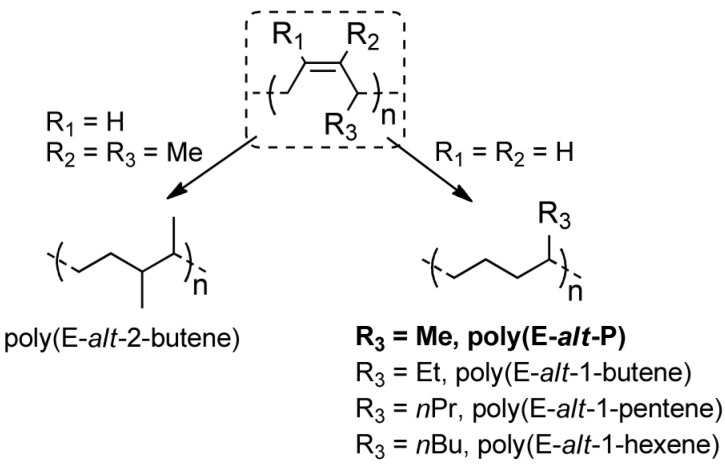
Saturated α-olefin (co)polymers which, at least in principle, can be obtained by hydrogenation of *cis*-1,4 poly(1,3-diene)s. In bold the alternating E/P copolymer object of the present investigation. Poly(E-*alt*-2-butene) can be also obtained with R_1_ = R_2_ = Me and R_3_ = H.

An alternative route for the synthesis of perfectly alternating ethylene/α-olefin copolymers in general, and of E/P copolymers in particular, is the one taking advantage of the hydrogenation of stereoregular poly(1,3-diene)s with 1,4-structure (*cis*-1,4 and *trans*-1,4 poly(1,3-pentadiene)s and poly(isoprene)s in case of E/P copolymers). In recent years we have introduced novel catalyst systems, based on different types of transition metal and lanthanide complexes with various ligands (e.g., phosphines, imines, pyridinimines, ketoimines) and methylaluminoxanes [9,10,11,12,13], which allowed some of us to synthesize and completely characterize highly stereoregular poly(1,3-diene)s with different structures (e.g., iso- and syndiotactic *cis*-1,4, *trans*-1,4, 1,2, 3,4) from various substituted 1,3-butadienes (e.g., butadiene, 2,3-dimethyl-1,3-butadiene, isoprene, (*E*)- and (*Z*)-1,3-pentadiene, (*E*)-2-methyl-1,3-pentadiene, (*E*)-3-methyl-1,3-pentadiene, 4-methyl-1,3- pentadiene, (*E*)- and (*Z*)-1,3-hexadiene, 5-methyl-1,3-hexadiene, (*E*,*E*)-2,4-hexadiene, (*E*)-1,3- heptadiene, (*E*)-1,3-octadiene) [14,15,16,17,18,19,20,21,22,23,24,25,26,27,28]. We decided to examine the hydrogenation of these highly stereoregular poly(1,3-diene)s since, at least in principle, it could allow one to get through the hydrogenation process: (*i*) highly stereoregular iso- and syndiotactic poly(α-olefin)s from highly stereoregular 1,2 and 3,4 poly(1,3-diene)s (Scheme 1); and (*ii*) perfectly alternating ethylene/α-olefin copolymers from highly stereoregular *cis*-1,4 and *trans*-1,4, iso- and syndiotactic, poly(1,3-diene)s (Scheme 2), which in some cases are unlikely to the obtainable through the simple stereospecific homo- and co-polymerization of the corresponding monomers. We have recently reported the synthesis and characterization of: (*i*) isotactic poly((*R*,*S*)-3-methyl-1-pentene) obtained by hydrogenation of isotactic 1,2 poly((*E*)-3-methyl-1,3-pentadiene) [29,30], and (*ii*) ethylene/2-butene copolymers obtained by hydrogenation of some *cis*-1,4 and *trans*-1,4 highly stereoregular poly(1,3-diene)s [31]. 

In the present paper, we report the preparation and characterization of perfectly alternating ethylene/propylene iso- and syndiotactic copolymers, obtained by hydrogenation of the following three highly stereoregular poly(1,3-diene)s: isotactic *cis*-1,4 poly(1,3-pentadiene), syndiotactic *cis*-1,4 poly(1,3-pentadiene), and *cis*-1,4 poly(isoprene).

## 2. Results and Discussion

The highly stereoregular poly(1,3-diene)s on which this study focused were: syndiotactic *cis*-1,4 poly(1,3-pentadiene) [hereafter named *s*P(EP)], isotactic *cis*-1,4 poly(1,3-pentadiene) [*i*P(EP)] and *cis*-1,4 poly(isoprene) (PI). *i*P(EP) and PI were obtained by polymerizing (*E*)-1,3-pentadiene and isoprene, respectively, with the ternary catalyst system AlEt_2_Cl/Nd(OCOC_7_H_15_)_3_/Al*i*Bu_3_, as described in [21,32,33], respectively. The *s*P(EP) was synthesized with catalyst CoCl_2_(P*^t^*Bu_2_Me)_2_/MAO as described in [34]. The polymerization data are summarized in Table 1.

The hydrogenation of the above polymers was then examined. There are different methods to hydrogenate poly(1,3-diene)s which involve both catalytic and non-catalytic ways [35]. Catalytic hydrogenation is the conventional one, but it has some drawbacks such as: (*i*) the high cost of the equipment, (*ii*) the use of expensive hydrogenation conditions, which are mostly associated with high pressure reactors and expensive catalyst, and (*iii*) the low efficiencies resulting from limited solubility of the reagents. Alternatively, the hydrogenation of unsaturated polymers by a non-catalytic way, in which the reaction is promoted by diimide (diazene, NH=NH), has been shown to be an attractive process and extremely efficient in the case of 1,3-diene polymers [36,37]. 

**Scheme 3:**
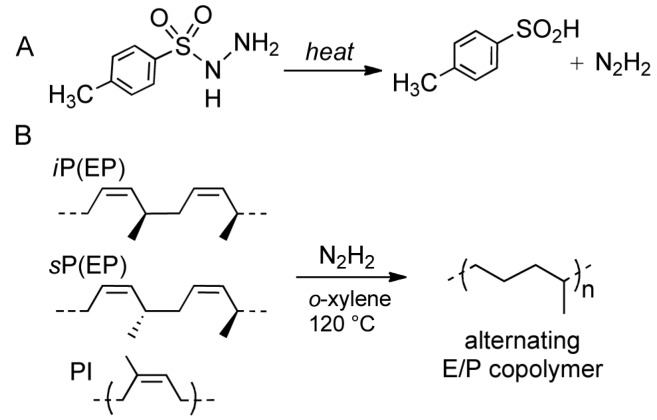
Scheme of hydrogenation of iso- and syndiotactic cis-1,4 poly(1,3-pentadiene) (iP(EP) and sP(EP)), and *cis*-1,4 poly(isoprene) (PI). *Reactions*: (**A**) diimide generation by thermal decomposition of TSH and (**B**) hydrogenation of unsaturated polymers with diimide.

In this work, we exploited the non-catalytic hydrogenation of the target stereoregular poly(1,3-diene)s with diimide in homogeneous conditions at 120 °C in *o*-xylene (Scheme 3). Diimide was generated in situ through the thermolysis of p-toluenesulfonic acid (TSH) [38]. The hydrogenation process converted iso- and syndiotactic *cis*-1,4 poly(1,3-pentadiene)s and *cis*-1,4 poly(isoprene) into perfectly alternating E/P copolymers (Scheme 3), having iso- or syndiotactic structures depending on the starting unsaturated polymer. Hereafter, we will name the alternating E/P copolymers from the hydrogenation of the corresponding 1,3-diene polymers as H-*s*P(EP), H-*i*P(EP) and H-PI, respectively. 

**Figure 1:**
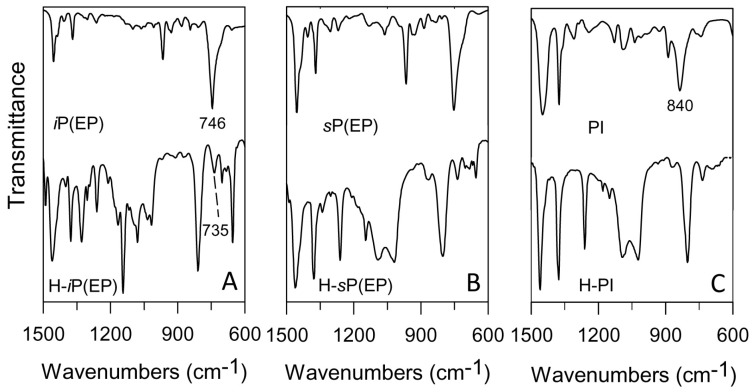
FTIR spectra of (**A**) isotactic *cis*-1,4 poly(1,3-pentadiene) (top) and its saturated polymers (bottom), (**B**) syndiotactic *cis* -1,4 poly(1,3-pentadiene) (top) and its saturated polymers (bottom) and (**C**) *cis*-1,4 poly(isoprene)(top) and its saturated polymers (bottom).

**Figure 2:**
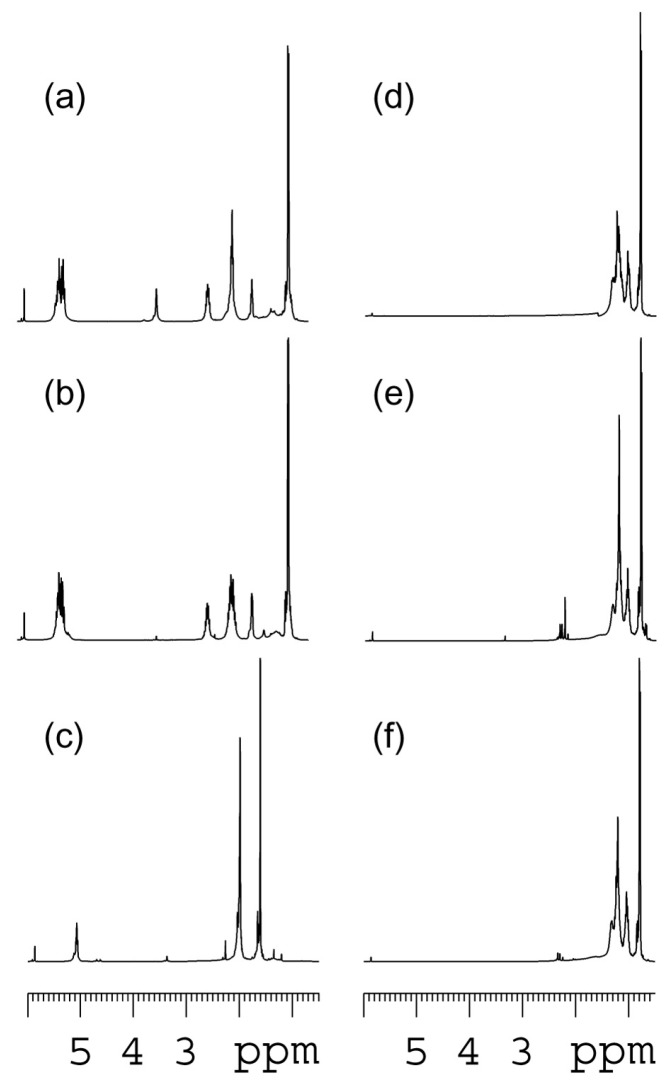
^1^H-NMR spectra (C_2_D_2_Cl_4_, 103 °C, HMDS) of the unsaturated *i*P(EP) (**a**), *s*P(EP) (**b**) and (PI) (**c**), and saturated H-*i*P(EP) (**d**), H-*s*P(EP) (**e**) and H-PI (**f**).

The complete hydrogenation of the diene polymers was confirmed by comparison of FTIR and ^1^H-NMR spectra of the starting *cis*-1,4 poly(1,3-diene)s and of the corresponding hydrogenated products (Figure 1 and Figure 2, respectively).

The typical bands observed at 746 cm^−1^ in the FTIR spectra of *cis*-1,4 poly(1,3-pentadiene)s and 840 cm^−1^ in the FTIR spectra of *cis*-1,4 poly(isoprene), ascribed to the out-of-plane vibration of the hydrogen atoms adjacent to the double bond in a *cis*-1,4 unit, are completely absent in the FTIR spectra of the corresponding saturated polymers; besides, a new band at 735 cm^−1^ was observed in the FTIR spectra of the hydrogenated polymers, ascribed to the vibration of a –CH_2_– unit, typical of saturated polyolefins (Figure 1).

The ^1^H-NMR spectra of sP(EP), iP(EP), PI and those of the corresponding hydrogenated products H-*s*P(EP), H-*i*P(EP) and H-PI, are shown in Figure 2. As it is clearly evident the peaks in the olefinic region (from 5.2 to 5.4 ppm), observed in the ^1^H-NMR spectra of the diene polymers, and due to the olefinic hydrogen atoms, are not observed in the ^1^H-NMR spectra of the hydrogenated polymers, confirming indeed the complete hydrogenation of the diene polymers. 

**Figure 3:**
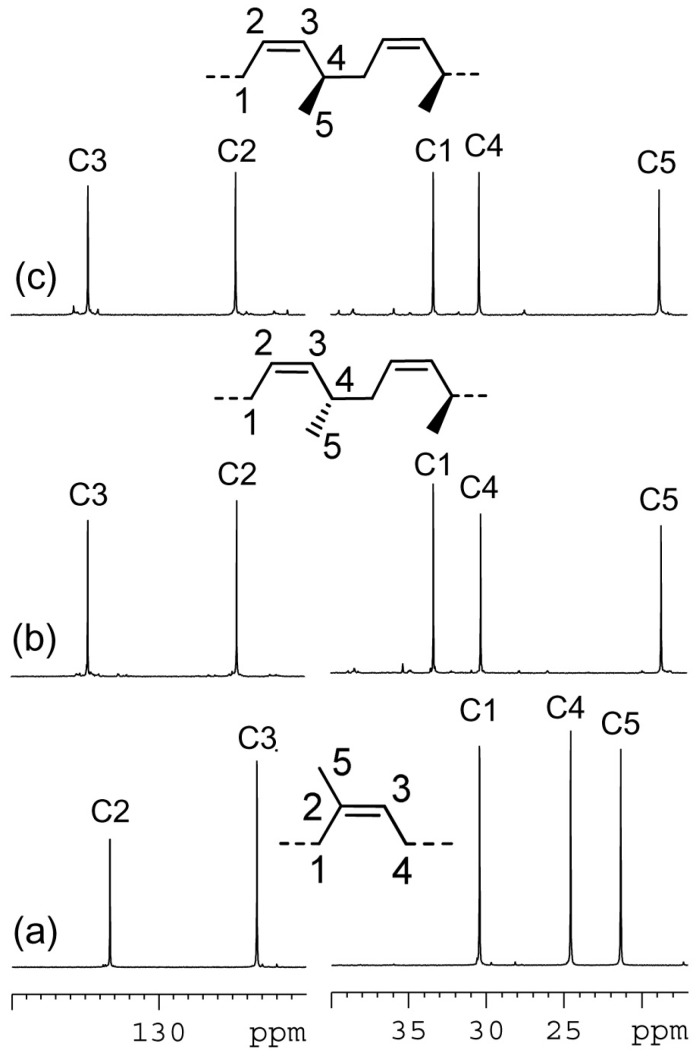
^13^C-NMR spectra (C_2_D_2_Cl_4_, 103 °C, HMDS) of the unsaturated polymer: (**a**) PI, (**b**) *s*P(EP) and (**c**) *i*P(EP).

The structure and tacticity of the resulting E/P copolymers were investigated by means of ^1^H and ^13^C-NMR, 2D NMR experiments [*i.e.*, Heteronuclear Single Quantum Correlation (HSQC)] and X-ray diffraction analysis. Figure 3 shows the ^13^C-NMR spectra of the diene polymers; the peaks were assigned as already reported [39].

**Figure 4:**
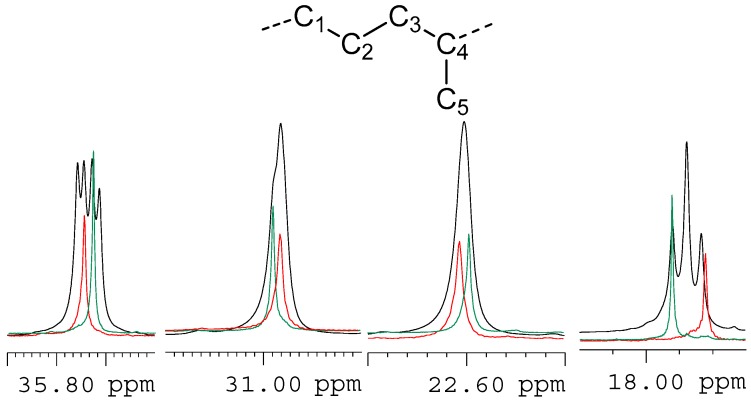
^13^C-NMR spectra (C_2_D_2_Cl_4_, 103 °C, HMDS) of the hydrogenated polymers: H-PI in black, H-*s*P(EP) in red, and H-*i*P(EP) in green.

**Figure 5:**
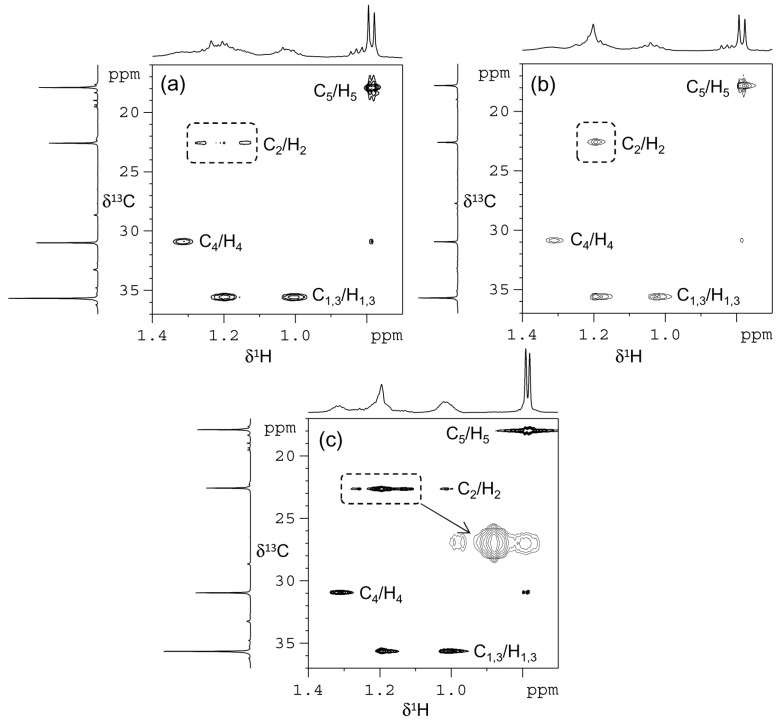
^1^H-^13^C HSQC of H-*i*P(EP) (**a**), H-*s*P(EP) (**b**) and H-PI (**c**), at 330 K in C_2_D_2_Cl_4_.

The ^13^C-NMR spectra of the hydrogenated polymers (E/P copolymers) are shown in Figure 4 and exhibit four major resonances around 17.9 (C5), 22.6 (C2), 31.0 (C4), and 35.7 (C1,C3) ppm likely corresponding to the four unique signals of a perfectly alternating E/P copolymer structure. The chemical shifts are very close for the three hydrogenated polymers so that, on the basis of the ^13^C-NMR spectra only, it is not possible to distinguish the tacticity of the copolymers obtained with the hydrogenation reaction. In principle, however, since the hydrogenation reaction in the case of poly(1,3-pentadiene)s does not lead to the formation of new asymmetric carbon atoms, it is reasonable to assume that the tacticity of the diene polymer precursors is maintained in the alternating resulting E/P copolymers, that is isotactic for H-*i*P(EP) and syndiotactic for H-*s*P(EP). This assumption was confirmed by means of the two-dimensional correlation spectroscopy, HSQC experiment, as the presence of a cross peak is indicative of protons and carbons directly linked through ^1^J_CH_, and was able to give information about the different tacticity of the E/P copolymers. Results of these experiments are shown in Figure 5. Specifically, the signal at about 22.6 ppm, corresponding to the Sββ methylene carbon, has proved to be diagnostic of the tacticity of the copolymer, as it is influenced by the arrangement of the methyl substituents, although not adjacent to the carbon atom bearing the methyl.

In the HSQC spectrum of the product of the hydrogenation of isotactic *cis*-1,4 poly(1,3-pentadene) (Figure 5a, *i*P(EP)), the C2 carbon at δ = 22.59 ppm correlates with two cross peaks at δ_H_ = 1.25 and 1.14 ppm, respectively. This is indicative of a methylene to which two magnetically non-equivalent protons are linked, as they display two different resonance frequencies “like in a different environment”, thus suggesting that the E/P copolymer obtained by hydrogenation of *i*P(EP) has an isotactic structure [40,41].

In the case of the E/P copolymer (Figure 5b) obtained by hydrogenation of *s*P(EP), the methylene at δ = 22.61 ppm showed a single correlation peak at δ_H_ = 1.19 ppm, meaning a linking with two magnetic equivalent protons, in agreement with the syndiotactic structure of H-*s*P(EP). Finally, for the E/P copolymer obtained by hydrogenation of PI, the 2D HSQC experiment suggests the coexistence of isotactic and syndiotactic stereoregularities (Figure 5c). The syndiotacticity (associated to the single cross peak for δ_C_ = 22.6 ppm, δ_H_ = 1.19 ppm) seems to be slightly preferred with respect to the isotacticity (two cross peaks for δ_C_ = 22.6 ppm, δ_H_ = 1.13 and 1.27 ppm), being the single cross peak intensity more pronounced in the 2D spectrum. Once the tacticity was established, the peaks multiplicity observed for the ^13^C resonances in the ^13^C-NMR spectrum of H-PI (Figure 4, black line; 35.72, 35.69, 35.66, 35.63 ppm for C1/C3 carbons; 17.92, 17.88, 17.84 for C5 carbon; some enlargement of the peaks at 22.60 and 30.96 ppm corresponding to carbons C2 and C4, respectively), could be reasonably related to the sensitivity of the carbon atoms up to triad level (in case of C5) and tetrads level (C1/C3 carbons).

**Figure 6:**
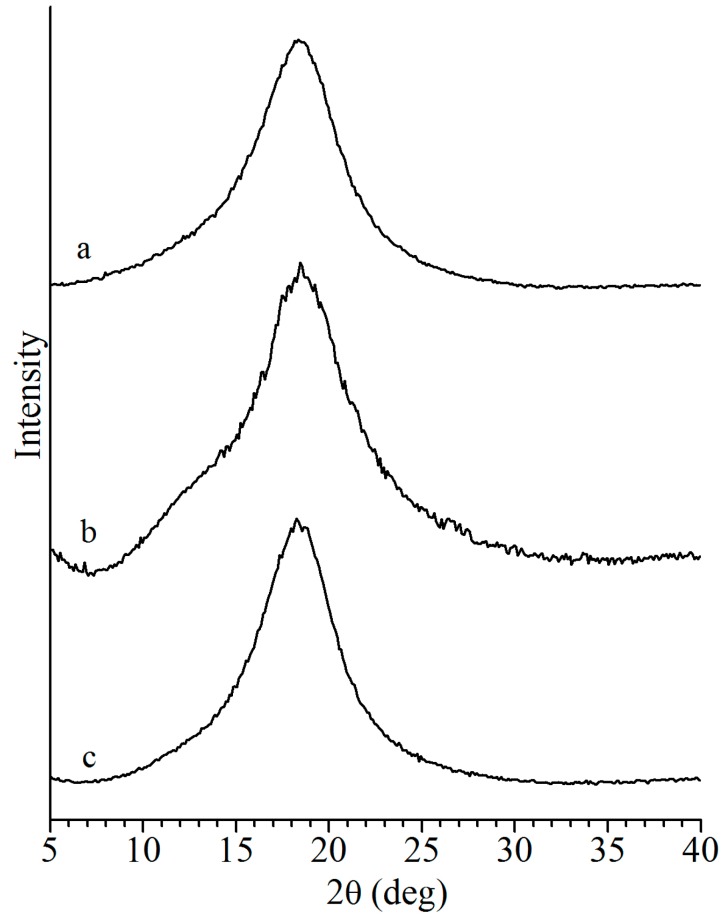
X-ray powder diffraction profiles of as-prepared samples of E/P alternating copolymers corresponding to the hydrogenated polymers H-*i*P(EP) (**a**), H-*s*P(EP) (**b**), and H-PI (**c**).

The X-ray powder diffraction profiles of the hydrogenated polymers H-*i*P(EP), H-*s*P(EP), and H-PI are shown in Figure 6. It is apparent that all samples show broad diffraction profiles with an absence of Bragg reflections, indicating that all samples are amorphous. The diffraction profiles do not change upon thermal treatments and the samples do not crystallize even after annealing at relatively high temperatures or upon aging at room and low temperatures. Moreover, in the case of sample *i*P(EP), that can be stretched at relatively high deformations, crystallization does not take place by stretching up to high degrees of deformation and even keeping the sample under tension at low temperature for long time. The NMR data of Figure 4 and Figure 5 as well as the diffraction data of Figure 6 indicate that the E/P alternating copolymers have a regular stereochemical structure, isotactic and syndiotactic for H-*i*P(EP) and H-*s*P(EP), respectively, and a statistical atactic structure for H-PI; the regular copolymers are however not able to crystallize. 

**Figure 7:**
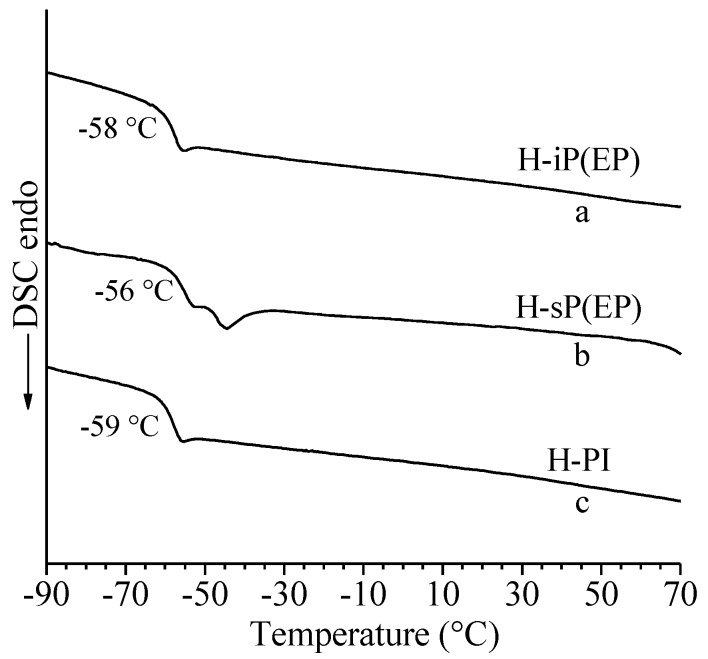
DSC heating curves, recorded at 10 °C/min, of as-prepared samples of alternating E/P copolymers corresponding to the hydrogenated polymers H-*i*P(EP) (**a**), H-*s*P(EP) (**b**), and H-PI (**c**).

The DSC heating curves of samples H-*i*P(EP), H-*s*P(EP) and H-PI are shown in Figure 7. According to the X-ray diffraction profiles of Figure 6, all DSC curves show only a glass transition at about −60 °C and the absence of any endothermic signal (Table 2). Only the glass transition at the same temperatures is observed in the successive cooling scans with absence of exothermic signals.

**Figure 8:**
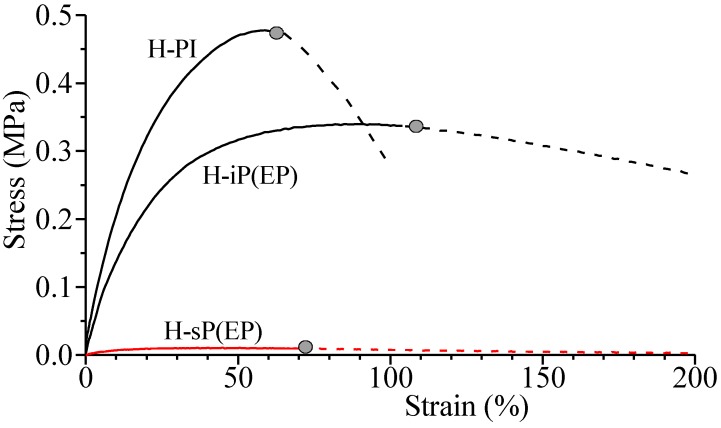
Stress-strain curves recorded at room temperature of compression-molded films of alternating E/P copolymers corresponding to the hydrogenated polymers H-*i*P(EP), H-*s*P(EP), and H-PI. The dashed portions of the stress-strain curves indicate the deformation at which viscous flow without breaking occurs.

The mechanical properties under tensile deformation of the three E/P alternating copolymers of different stereochemical structure have also been studied. The stress-strain curves recorded at room temperature of compression-molded films of the hydrogenated polymers are shown in Figure 8. According to the absence of crystallinity, all samples show low values of the Young modulus (0.6–1.0 MPa) and of the stress at any strain and viscous flow already at relatively low deformations. The differences in the stress-strain curves are mainly related to the different molecular masses of the three alternating copolymers. Higher values of the stress are, indeed, shown by the sample H-PI of higher molecular mass (Figure 8).

## 3. Materials and Methods 

### 3.1. Materials

Manipulations of air- and/or moisture-sensitive materials were carried out under an inert atmosphere using a dual vacuum/nitrogen line and standard Schlenk-line techniques. Toluene (≥99.7% pure, Aldrich, Milano, Italy) and heptane (≥99% pure, Aldrich) were refluxed over Na for about 8 h and then distilled and stored over molecular sieves under nitrogen. *o*-Xylene (Aldrich, anhydrous grade), *p*-Toluenesulfonylhydrazide (TSH, Aldrich), deuterated solvent for NMR measurements (C_2_D_2_Cl_4_) (Cambridge Isotope Laboratories, Inc., Tewksbury, MA, USA), diethylaluminum chloride (AlEt_2_Cl) (97% pure Fluka-Aldrich, Milano, Italy), triisobutylaluminum (Al(*i*Bu)_3_) (98% pure, Aldrich), methylaluminoxane (MAO) (10 wt% solution in toluene, Aldrich) and Nd(OCOC_7_H_15_)_3_ (Strem Chemicals, Bischheim, France) were used as received. (*E*)-1,3-pentadiene (96% pure, Fluka) and isoprene (98%, Aldrich,) were refluxed over calcium hydride for about 4 h, then distilled trap-to-trap, and stored under nitrogen. Isotactic *cis*-1,4-poly(*E*-1,3-pentadiene) and *cis*-1,4 poly(isoprene) were synthesized with the catalyst AlEt_2_Cl/(NdOCOC_7_H_15_)/Al*i*Bu_3_ as described in reference 21 and 32/33, respectively; syndiotactic *cis*-1,4 poly(*E*-1,3-pentadiene) was synthesized with the catalyst CoCl_2_(P*^t^*Bu_2_Me)_2_/MAO as described in [34].

### 3.2. Hydrogenation Procedure

The hydrogenation was carried out in a round-bottom flask equipped with a reflux condenser, a nitrogen inlet port, and a temperature controller. Typically, the specified amount of the diene polymer was dissolved in *o*-xylene. The mixture was continuously stirred at room temperature until the polymer was completely dissolved. TSH was then added and the mixture was refluxed by slowly heating to 120 °C. After 3 days the mixture was allowed to cool spontaneously to room temperature and TSH was added. This operation is repeated once again. Upon completion of the reaction, the hydrogenated sample was hot-filtered, the volume of the filtered solution was reduced under vacuum, and the dissolved polymer precipitated with methanol and collected by filtration. The polymer was dried under vacuum at room temperature, and then it was extracted with acetone through a Soxhlet method for 10 h in order to remove any excess TSH and by products originating from TSH decomposition. The residual polymer was finally dried under vacuum, dissolved in toluene, precipitated into methanol, and dried again under vacuum at room temperature to constant weight. Specifically, for each polymer sample the conditions were: 

#### 3.2.1. Hydrogenation of Isotactic *cis*-1,4 poly(1,3-pentadiene)

Polymer 2.9 g; xylene, 300 mL; first addition of THS, 20.0 g (1.07 × 10^−1^ mol); second and third addition of THS, 30.0 g (1.87 × 10^−1^ mol).

#### 3.2.2. Hydrogenation of Syndiotactic *cis*-1,4 poly(1,3-pentadiene) 

Polymer 0.5 g; xylene, 70 mL; first addition of THS, 9.0 g (5.0 × 10^−2^ mol); second and third addition of THS, 10.0 g (5.56 × 10^−2^ mol).

#### 3.2.3. Hydrogenation of *cis*-1,4 poly(isoprene)

Polymer 0.5 g; xylene, 100 mL; first addition of THS, 8.0 g (4.44 × 10^−2^ mol); second and third addition of THS, 9.0 g (5.0 × 10^−2^ mol).

#### 3.3. Characterization

^13^C- and ^1^H-NMR measurements were carried out on an Avance 400 spectrometer (Bruker, Milano, Italy) The spectra were obtained in C_2_D_2_Cl_4_ at 103 °C (hexamethyldisiloxane, HMDS, as internal standard). The concentration of polymer solutions was about 10 wt %. 13C parameters were: spectral width 17 kHz; 90° pulse 11.0 μs PL1 −5.0 dB, with a delay of 16 s. The 2D spectra were acquired on a Bruker DMX 600 MHz instrument, (14.1 T), at 330 K. The g-HSQC experiment, was performed by considering a coupling constant ^1^J_CH_ = 130 Hz; data matrix 2 K × 256; number of scans128, 90° pulse calculated for each sample, PL1 −2.2 dB. Molecular weight averages (*M*_w_) and molecular weight distribution (*M*_w_/*M*_n_) were obtained by a high temperature GPCV2000 size exclusion chromatography (SEC) system (Waters, Milano, Italy) using two online detectors: a differential viscometer and a refractometer. The experimental conditions consisted of three PL Gel Olexis columns, *o*-dichlorobenzene as the mobile phase, 0.8 mL min^−1^ flow rate, and 145 °C temperature. Universal calibration of the SEC system was performed using eighteen narrow *M*_w_/*M*_n_ polystyrene standards with molar weights ranging from 162 to 5.6 × 10^6^ g mol^−1^. For the analysis, about 12 mg of the polymer was dissolved in 5 mL of *o*-dichlorobenzene with 0.05% of BHT as the antioxidant. 

X-ray powder diffraction profiles were obtained with Ni filtered CuKα radiation with an automatic diffractometer X-Pert by Panalytical (Westborough, MA, USA). Thermal analysis was performed with a DSC30/2285 differential scanning calorimeter (Mettler, Milano, Italy) equipped with a liquid nitrogen cooling system for measurements at low temperature. The scans were recorded in flowing nitrogen atmosphere at heating or cooling rates of 10 °C min^−1^. Compression molded samples were prepared by heating the dry-precipitated powders at 120 °C for 10 min under a press at very low pressure and cooling to room temperature by circulating cold water inside the press plates. 

Mechanical tests have been performed at room temperature on compression-molded films with a universal mechanical tester Zwicky by ZwickRoell (Genova, Italy), following the standard test method for tensile properties of thin plastic sheeting ASTM D882-83. Rectangular specimens 10 mm long, 5 mm wide and 0.3 mm thick have been stretched up to the break or up to a given deformation ε = 100 × (*L*_f_ − *L*_0_)/*L*_0_, where *L*_0_ and *L*_f_ are the initial and final lengths of the specimen, respectively. Two benchmarks have been placed on the test specimens and used to measure elongation. The ratio between the drawing rate and the initial length was fixed equal to 0.1 mm/(mm × min) for the measurement of Young’s modulus and 10 mm/(mm × min) for the measurement of stress-strain curves. The reported stress-strain curves and the values of the mechanical properties are averaged over at least five independent experiments. 

## 4. Conclusions

Perfectly alternating ethylene/propylene copolymers having isotactic, syndiotactic and atactic structures have been obtained by means of hydrogenation of highly stereoregular isotactic *cis*-1,4 poly(1,3-pentadiene), syndiotactic *cis*-1,4 poly(1,3-pentadiene) and *cis*-1,4-poly(isoprene), respectively. The copolymers were fully characterized by NMR (^1^H, ^13^C and 2D), XRD, and their thermal and mechanical properties were investigated. Despite their high stereoregularity, as clearly evidenced by the NMR analysis, all the copolymers were found to be amorphous, exhibiting low mechanical properties.

## Figures and Tables

**Table:** 

**polymer/ppm**	**C1**	**C2**	**C3**	**C4**	**C5**
**PI**	30.44	123.35	133.30	24.60	21.38
***s*P(EP)**	33.37	124.62	134.75	30.31	18.73
***i*P(EP)**	33.38	124.73	134.72	30.42	18.84

**Table:** 

**Polymer/ppm**	**C1/C3**	**C2**	**C4**	**C5**
**H-PI**	35.72	22.60	30.96	17.92
	35.69			17.88
	35.66			17.84
	35.63			
**H*-s*P(EP)**	35.69	22.61	30.96	17.82
**H*-i*P(EP)**	35.66	22.59	30.98	17.92

**Table 1:** Polymerization of (*E*)-1,3-pentadiene and isoprene ^1^.

Sample	Monomer	Catalyst	Time	Yield	*cis*-1,4 ^2^	*T*_g_^3^
			(min)	(%)	(%)	(°C)
*i*P(EP)	(*E*)-1,3-pentadiene	AlEt_2_Cl/Nd(OCOC_7_H_15_)_3_/Al*^i^*Bu_3_	60	87	≥90	−66
*s*P(EP)	(*E*)-1,3-pentadiene	CoCl_2_(P*^t^*Bu_2_Me)_2_/MAO	144	69	≥99	−66
PI	isoprene	AlEt_2_Cl/Nd(OCOC_7_H_15_)_3_/Al*^i^*Bu_3_	30	100	≥97	−65

^1^ polymerization conditions: monomer (2 mL); heptane (total volume 16 mL) (or toluene in the case of *s*P(EP)), Co or Nd complex, 2 × 10^−5^ mol; temperature, 22 °C; ^2^ Percentage of *cis*-1,4 units, determined by NMR analysis; ^3^ determined by DSC.

**Table 2:** Values of average molecular mass (*M*_w_), molecular weight distribution (*M*_w_/*M*_n_) and glass transition temperature (*T*_g_) of the alternated E/P copolymers obtained by hydrogenation of the corresponding poly(1,3-diene)s.

E/P Copolymers	Corresponding Poly(1,3-diene)	*M*_w_^1^ (×10^−3^)	*M*_w_/*M*_n_*^1^*	*T*_g_^2^ (°C)
H-*i*P(EP)	isotactic *cis*-1,4 poly(1,3-pentadiene)	194.8	2.8	−58
H-*s*P(EP)	syndiotactic *cis*-1,4-poly(1,3-pentadiene)	60.5	3.1	–56
H-PI	*cis*-1,4 poly(isoprene)	249.3	2.8	–59

^1^ determined by SEC; ^2^ determined by DSC.
